# Anxiolytic Effect of Exogenous Ketone Supplementation Is Abolished by Adenosine A1 Receptor Inhibition in Wistar Albino Glaxo/Rijswijk Rats

**DOI:** 10.3389/fnbeh.2018.00029

**Published:** 2018-02-22

**Authors:** Zsolt Kovács, Dominic P. D'Agostino, Csilla Ari

**Affiliations:** ^1^Savaria Department of Biology, Eötvös Loránd University (ELTE), Budapest, Hungary; ^2^Department of Molecular Pharmacology and Physiology, Metabolic Medicine Research Laboratory, Morsani College of Medicine, University of South Florida, Tampa, FL, United States; ^3^Institute for Human and Machine Cognition, Ocala, FL, United States; ^4^Department of Psychology, Hyperbaric Neuroscience Research Laboratory, University of South Florida, Tampa, FL, United States

**Keywords:** exogenous ketone supplement, ketosis, anxiety, EPM, adenosine, WAG/Rij rats

## Abstract

Anxiety disorders are one of the most common mental health problems worldwide, but the exact pathophysiology remains largely unknown. It has been demonstrated previously that administration of exogenous ketone supplement KSMCT (ketone salt/KS + medium chain triglyceride/MCT oil) by intragastric gavage for 7 days decreased the anxiety level in genetically absence epileptic Wistar Albino Glaxo/Rijswijk (WAG/Rij) rats. To investigate the potential role of the adenosinergic system in the pathomechanism of anxiety we tested whether the inhibition of adenosine A_1_ receptors (A_1_Rs) influence the anxiolytic effect of the exogenous ketone supplement. As A_1_Rs may mediate such an effect, in the present study we used a specific A_1_R antagonist, DPCPX (1,3-dipropyl-8-cyclopentylxanthine) to test whether it modulates the anxiolytic effect of sub-chronically (7 days) applied KSMCT in the previously tested animal model by using elevated plus maze (EPM) test. We administered KSMCT (2.5 g/kg/day) alone by intragastric gavage and in combination with intraperitoneally (i.p.) injected of DPCPX in two doses (lower: 0.15 mg/kg, higher: 0.25 mg/kg). Control groups represented i.p saline and water gavage with or without i.p. DPCPX administration (2.5 g/kg/day). After treatments, the level of blood glucose and beta-hydroxybutyrate (βHB), as well as body weight were recorded. KSMCT alone significantly increased the time spent in the open arms and decreased the time spent in the closed arms, supporting our previous results. Injection of lower dose of DPCPX decreased, while higher dose of DPCPX abolished the effect of KSMCT administration on EPM. Blood βHB levels were significantly increased after administration of KSMCT, while DPCPX did not change the KSMCT induced increase in blood βHB levels. These results demonstrate that A_1_R inhibition modified (decreased) the anti-anxiety effect of KSMCT administration implying that the adenosinergic system, likely *via* A_1_Rs, may modulate the exogenous ketone supplement induced anxiolytic influence.

## Introduction

Anxiety disorders, such as panic disorder, post-traumatic stress disorder, and specific phobias, are one of the most common mental health problems worldwide, which disorders may be associated with impairment of quality of life (Li, 2012). Lifetime prevalence of anxiety disorders may be up to 14% worldwide while about 28% of U.S. population suffers anxiety-related disorders during their lifetime (Kessler et al., 2005; Garakani et al., 2006).

Our knowledge about the exact cause and pathomechanism of anxiety disorders is far from complete. It has been demonstrated that mainly glutamatergic, serotoninergic, GABAergic and, based on recent results, adenosinergic systems as well as different brain structures (e.g., amygdala and hippocampus) contribute to the pathophysiology of anxiety (Kakui et al., 2009; Masino et al., 2009; Li, 2012; Dias et al., 2013). Indeed, for example benzodiazepines, D-cycloserine, selective serotonin reuptake inhibitors and serotonin receptor 5-HT1A agonists (e.g., buspirone) are effective for alleviating anxiety symptoms (Engin and Treit, 2008; Norberg et al., 2008; Li, 2012; Dias et al., 2013). Although the exact role of adenosinergic system in the pathomechanism of anxiety is poorly understood, it has been previously suggested that adenosine may reduce anxiety (Jain et al., 1995; Johansson et al., 2001; Masino et al., 2009, 2012), which approach may lead to a new therapeutic approach.

Ketone bodies, such as beta-hydroxybutyrate (βHB) and acetoacetate (AcAc) cross the blood brain barrier, enter into the cells by monocarboxylic transporters (MCT1-4) and provide a source of energy for the CNS. Ketosis may contribute to adenosine triphosphate (ATP) production by metabolism of ketone bodies to acetyl-CoA (Veech, 2004; Yudkoff et al., 2007; Achanta and Rae, 2017), but βHB and AcAc are more than just energy metabolites. They have a broad range of signaling effects in the body that can enhance overall metabolism, decrease inflammatory processes, suppress oxidative stress, and regulate receptor and ion channel functions (Bough and Rho, 2007; Newman and Verdin, 2014; Rogawski et al., 2016). Emerging data has shown that therapeutic ketosis may improve the symptoms of neurological diseases, such as Parkinson's disease, Alzheimer's disease, schizophrenia, glucose transporter 1-deficiency syndrome, anxiety, autism, depression, and epilepsy in animal models (Hashim and VanItallie, 2014; Bostock et al., 2017; Cheng et al., 2017; Rho, 2017). It has also been demonstrated that administration of exogenous ketone supplements (normal food + ketone supplements and/or medium chain triglyceride/MCT), such as ketone ester (KE), ketone salt (KS) or their combination with MCT oil (e.g., KSMCT) induce rapid and sustained nutritional ketosis (D'Agostino et al., 2013; Poff et al., 2015; Ari et al., 2016; Kesl et al., 2016) and may therapeutically target central nervous system (CNS) diseases, such as epilepsy and anxiety in animal models (D'Agostino et al., 2013; Ari et al., 2016; Kovács et al., 2017). In addition, human pilot studies and case studies suggest that ketogenic diet and ketogenic supplement-induced ketosis is a potential therapeutic approach in the treatment of epilepsy, Alzheimer's disease, autism spectrum disorder, and schizophrenia (Pacheco et al., 1965; Evangeliou et al., 2003; Stafstrom and Rho, 2012; Newport et al., 2015). However, contradicting results have also been demonstrated regarding the effect of ketosis on CNS diseases. For example, a study showed that the Atkins diet may worsen panic disorder likely through ketosis and modulation of serotoninergic system (Ehrenreich, 2006; Li, 2012). On the other hand, ketosis-based therapy has been found efficient (Rho et al., 2002; Likhodii et al., 2003; Minlebaev and Khazipov, 2011; Kim et al., 2015), while in other studies it was found inefficient against epileptic seizures in different seizure models suggesting that the effects of ketosis on CNS diseases may be model-, dose-, method- and regimen-dependent (Thavendiranathan et al., 2000; Bough et al., 2002). In spite of results suggesting that ketone bodies may exert their effects through modulation of GABAergic, glutamatergic, and adenosinergic system (Yudkoff et al., 2007; Masino et al., 2011; Kovács et al., 2017; Rho, 2017), the specific mechanism of exogenous ketone supplements/nutritional ketosis on CNS diseases are largely unknown. Therefore, further studies are necessary to better understand the exact effects and signal transduction mechanisms potentially induced by exogenous ketone supplements under different circumstances and in different animal models.

It has been demonstrated, that sub-chronic (7 days) oral administration of exogenous ketone supplement KSMCT decreased not only the absence epileptic activity (number of spike-wave discharges: SWDs) (Kovács et al., 2017), but also anxiety level on elevated plus maze (EPM) (Ari et al., 2016) in correlation with increased level of βHB in genetically absence epileptic Wistar Albino Glaxo/Rijswijk (WAG/Rij) rats (Coenen and Van Luijtelaar, 2003). It was also demonstrated that blockade of adenosine A_1_ receptors (A_1_Rs) abolished the alleviating effect of KSMCT on absence epileptic seizures (Kovács et al., 2017), suggesting that the ketone-induced anxiolytic effect may be mediated, in part, through the adenosinergic system. Indeed, adenosinergic system has been proposed to modulate anxiety-like behavior in previous studies (Klein et al., 1991; Johansson et al., 2001; Giménez-Llort et al., 2002; Cunha et al., 2008).

EPM is a test based on the rodents' unconditioned fear of open spaces/heights (avoidance), proclivity toward enclosed, protected/dark spaces (approach) and their innate motivation to explore novel environments (Walf and Frye, 2007; Engin and Treit, 2008). Increased proportion of time spent on open arms and elevated number of entries to open arms measured by EPM may reflect reduction in anxiety level (Walf and Frye, 2007; Engin and Treit, 2008). In previous studies it has been shown that the three commonly used tests that are considered to be suitable to measure anxiety (light–dark choice, social interaction and EPM) revealed no significant difference in anxiety level between WAG/Rij and Wistar rats (Sarkisova et al., 2003), while EPM is considered a suitable method to assess the anxiety responses of different adenosine receptor agonists and antagonists (Jain et al., 1995; Florio et al., 1998). Therefore, in order to make our results comparable to our previous study (Ari et al., 2016), in the present study we used the same animal model (WAG/Rij rats) and behavioral test (EPM) to investigate whether inhibition of A_1_Rs by its specific antagonist DPCPX (1,3-dipropyl-8-cyclopentylxanthine) can modulate the effect of sub-chronically (for 7 days) applied KSMCT on anxiety-like behavior. To first confirm our previous results on the effect of KSMCT on anxiety level, βHB and glucose levels (Ari et al., 2016) we administered KSMCT (2.5 g/kg/day) alone by intragastric gavage in WAG/Rij rats. To examine whether A_1_Rs can modify the observed KSMCT-evoked changes on anxiety level, we used two doses (lower: 0.15 mg/kg and higher: 0.25 mg/kg) of DPCPX intraperitoneally (i.p.) in combination with KSMCT (2.5 g/kg/day, gavage). In addition, we investigated the effect of higher dose of i.p. DPCPX (0.25 mg/kg) alone on anxiety level.

We hypothesized that A_1_R inhibition may modify the anti-anxiety effect of KSMCT administration.

## Materials and methods

### Animals

All animal treatments and surgery procedures were carried out according to the local ethical rules, which are in conformity with the guidelines of the Hungarian Act of Animal Care and Experimentation (1998, XXVIII, section 243), European Communities Council Directive 24 November 1986 (86/609/EEC) and EU Directive 2010/63/EU on the use and treatment of animals in experimental laboratories. The experimental design was approved by the Animal Care and Experimentation Committee of the Eötvös Loránd University (Savaria University Center) and National Scientific Ethical Committee on Animal Experimentation (Hungary) under license number VA/ÉBNTF02/85-8/2016, and was compliant with the Ethics Codex of institution.

WAG/Rij male rats (*n* = 40; 8 months old, 305–340 g; breeding colony of WAG/Rij rats at Eötvös Loránd University, Savaria University Center, Szombathely, Hungary) were housed in groups 3-4 under standard laboratory conditions (12:12 h light-dark cycle: light was on from 08.00 AM to 08.00 PM; free access to water and food; air-conditioned room at 22 ± 2°C).

### Synthesis and application of ketone precursor KSMCT

Ketone salt (KS: Na^+^/K^+^ - βHB mineral salt) was mixed into a 50% solution supplying approximately 375 mg/g of pure βHB and 125 mg/g of Na^+^/K^+^ in a 1:1 ratio. Ketone salt was developed and synthesized in collaboration with Savind Inc. (D'Agostino et al., 2013; Kesl et al., 2016). Human food grade MCT oil (~60% caprylic triglyceride and ~40% capric triglyceride) was purchased from Now Foods (Bloomingdale, IL, USA), which was mixed with KS in a 1:1 ratio (KSMCT).

We demonstrated previously the tolerability and effectiveness of exogenous ketone supplement KSMCT given by intragastric gavage (*ad libitum* access to normal rat chow + 2.5 g/kg body weight KSMCT by intragastric gavage once/day), which dose induced and maintained ketosis (Ari et al., 2016; Kesl et al., 2016; Kovács et al., 2017) and did not generate side effects, such as diarrhea or death. Therefore, in this study, KSMCT of 2.5 g/kg/day dosage was administered daily by intragastric gavage for 7 days.

### Anxiety assay

EPM was used to assess anxiety-related behavior of the rats after 7 days of intragastric gavage of KSMCT. EPM is a plus-shaped apparatus, which consists of four, 11 cm wide and 50 cm long arms: two opposite arms are opened and two closed opposite arms were equipped with 40 cm high walls. The apparatus is elevated 80 cm above the floor. The square shaped center (intersection of the arms) of the apparatus was lit by about 70 Lux. EPM experiments were carried out between 12.00 and 14.00 h as was described previously (Ari et al., 2016). Briefly, rats were placed in the intersection of the four arms of the EPM, facing the open arm away from the experimenter. Similarly to our previous study, the behavior of animals on EPM was video recorded for 5 min and the following data was collected: time spent and number of entries made to open arms, closed arms and to the center zone. The apparatus was cleaned with 70% ethyl-alcohol following with tap water and finally, arms were dried with paper towel between rats. A blinded observer was present in the testing room separated from the EPM by a curtain while collecting data.

### Measuring of blood glucose and βHB levels

Blood glucose (mg/dl) and D-βHB (mmol/L) levels were measured following the EPM test from blood taken from the tail vein with a commercially available glucose and ketone monitoring system 60 min after gavage (Precision Xtra™, Abbott Laboratories, Abbott Park, IL, USA, Ari et al., 2016). This ketone monitoring system measures D-βHB only, thus, total blood ketone levels (D-βHB + L-βHB + AcAc + acetone) from contribution of racemic KS would be higher.

### Experimental design

**Figure 1:**
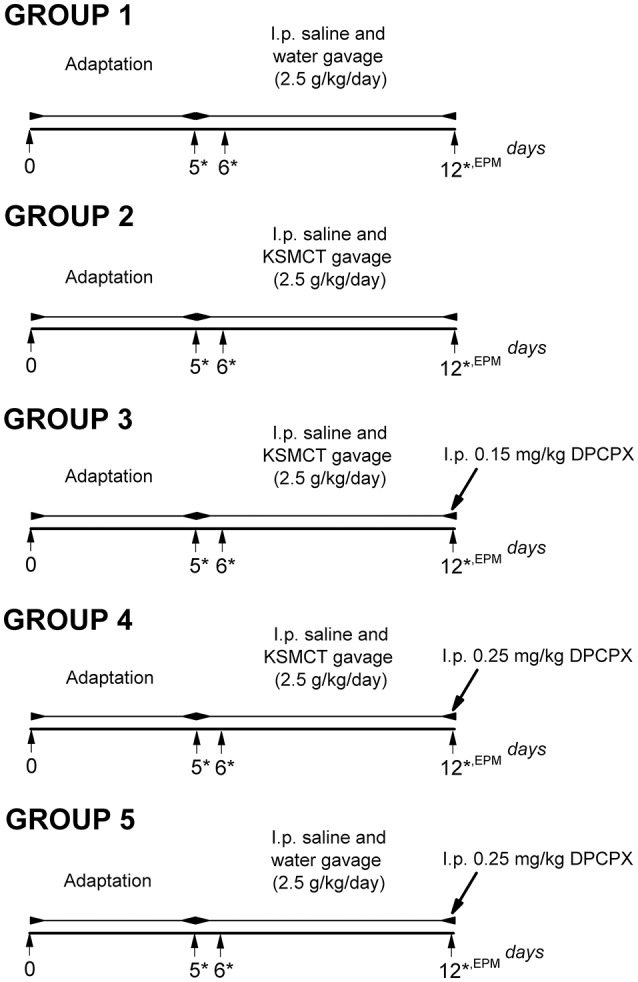
Experimental design of WAG/Rij rats (8 animals/groups). ^*^, days of blood glucose and beta-hydroxybutyrate (βHB) level measurements; DPCPX, 1,3-Dipropyl-8-cyclopentylxanthine; EPM, elevated plus maze; i.p., intraperitoneally; KSMCT, ketone salt/KS + medium chain triglyceride/MCT.

Rats were assigned into 5 groups (Figure 1) with 8 animals in each group. To adapt the animals to the intragastric gavage method and i.p. injections, we applied i.p. saline (0.5 ml/100 g) injection and water gavage (2.5 g/kg/day) on 5 consecutive days (between 1st and 5th day of the experiment: adaptation period) (group 1-5). After the adaptation period, in order to establish the effect of KSMCT on anxiety level, we administered i.p. 0.5 ml/100 g saline and 2.5 g/kg/day water (group 1; control) or 2.5 g/kg/day KSMCT (group 2) alone by gavage between 6th and 12th day of the experiments (Figure 1), followed by EPM on the 12th day.

In order to induce antagonism of A_1_Rs without changes in absence epileptic activity and to decrease putative side effects of DPCPX we used 0.15 mg/kg and 0.25 mg/kg DPCPX in combination with KSMCT (2.5 g/kg/day) because these DPCPX doses alone did not change the SWD number in WAG/Rij rats (Kovács et al., 2017, and unpublished, preliminary results).

To investigate the effect of the A_1_R antagonist DPCPX on KSMCT-evoked influence on EPM, after the adaptation period and 6-day treatment by i.p. 0.5 ml/100 g saline and KSMCT (2.5 g/kg/day) gavage, 0.15 mg/kg (group 3) or 0.25 mg/kg (group 4) DPCPX in 0.5 ml 10% dimethyl sulfoxide (DMSO)/100 g body weight were i.p. injected on the 7th day of KSMCT gavage (12th day of the experiment) (Figure 1) followed by EPM. It is interesting to note that 1–30% (v/v) DMSO solution have no effect on absence epileptic activity in WAG/Rij rats (Kovács et al., 2011a). To reveal the putative influence of A_1_R inhibition on EPM, before EPM assay, adaptation, i.p. saline injection and water gavage (2.5 g/kg/day; between 6th and 11th day of the experiment) as well as i.p. injection of 0.25 mg/kg DPCPX in 0.5 ml 10% DMSO/100 g body weight on the 7th day of water gavage (12th day of the experiment) were also conducted (group 5) (Figure 1). All i.p. injections (saline and DPCPX) were given 30 min prior to gavage (group 1–5).

To investigate the effect of KSMCT and DPCPX alone as well as in combination on blood glucose and βHB levels, we measured these blood parameters on the last day of adaptation period (5th day, baseline), and on the days of the 1st and the 7th treatments (on the 6th and 12th day of experiments; group 1–5) (Figure 1). The body weight of rats was also measured before treatments started (5th day of adaptation period: baseline) and after the last (7th) treatments (on the 12th day of experiments; group 1–5).

All results were presented as means ± standard error of the mean (S.E.M.), similarly to our previous work (Ari et al., 2016). We compared the effects of KSMCT and DPCPX alone and in combination on anxiety-related behavior, on blood βHB and glucose levels, as well as on body weight to control (group 1) and to baseline (5th day of adaptation period) levels. Data analysis was performed using GraphPad PRISM version 6.0a. Significance was determined by one-way or two-way analysis of variance (ANOVA) with Tukey's multiple comparisons test and Sidak's multiple comparisons test. Results were considered significant when *p* < 0.05 (Ari et al., 2016).

## Results

### EPM assay: effect of KSMCT and DPCPX on anxiety level

**Figure 2:**
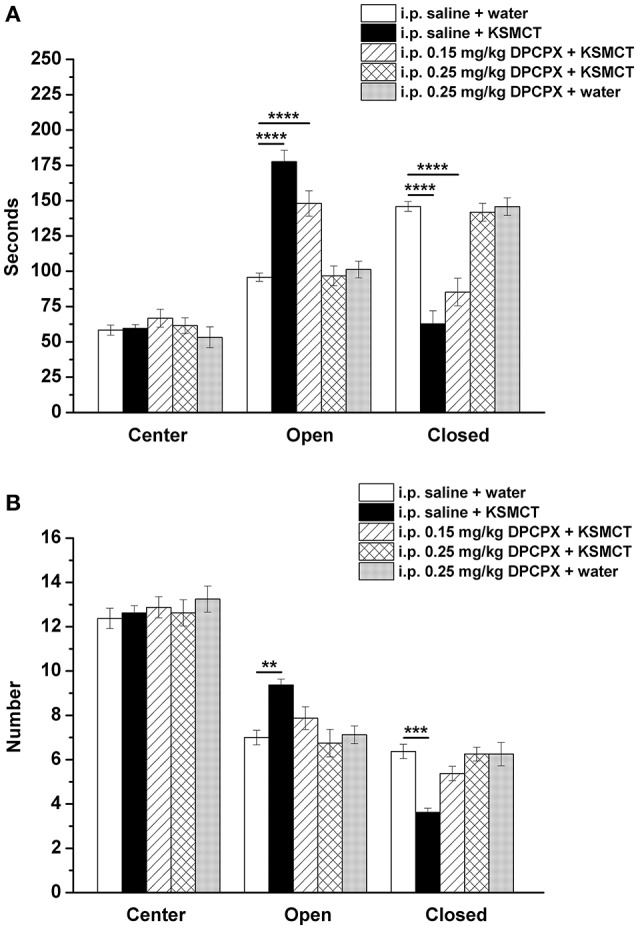
Effect of KSMCT (gavage, 2.5 g/kg/day, *n* = 8; i.p. saline + KSMCT) alone, KSMCT in combination with intraperitoneally (i.p.) injected two doses (0.15 mg/kg, *n* = 8; 0.25 mg/kg, *n* = 8) of DPCPX as well as higher dose of DPCPX (0.25 mg/kg, *n* = 8; DPCPX + water) on the time spent in the areas (center, open arms, and closed arms) **(A)** and number of entries to the areas **(B)** of EPM, compared to control animals (i.p. saline + water; *n* = 8). All results are shown as means ± standard error of the mean (S.E.M.). DPCPX, 1,3-Dipropyl-8-cyclopentylxanthine; i.p., intraperitoneally; KSMCT, ketone salt/KS + medium chain triglyceride/MCT; ^**^p < 0.01, ^***^p < 0.001, and ^****^p < 0.0001 level of significance.

Consistent with our previous study (Ari et al., 2016), after 7 days of oral gavage, KSMCT (2.5 g/kg/day) significantly increased the time spent in the open arms (*p* < 0.0001), whereas decreased the time spent in the closed arms (p < 0.0001; group 2) (Figures 1, 2A), compared to control (i.p. saline + water: group 1). Injection of both doses of i.p. DPCPX (0.15 mg/kg and 0.25 mg/kg) 30 min before the 7th application of KSMCT (Figure 1, group 3 and group 4) dose-dependently decreased (0.15 mg/kg DPCPX) and abolished (0.25 mg/kg DPCPX) the effect of KSMCT gavage on the time spent in the arms (Figure 2A; Table 1). In addition, i.p. 0.25 mg/kg DPCPX alone (group 5) did not change the time spent in the arms compared to control. Time spent in the center was not changed by the treatments compared to the control group (Figure 2A; Table 1).

**Table 1:** Time spent in the areas (center, open arms, and closed arms) and number of entries to the areas of EPM after different treatments are presented (8 animals/group).

**Areas of EPM and different treatments (the time spent in the areas, in seconds, see Figure 2A)**	**Mean/±S.E.M**.	**Significance/*q*-value**
**CENTER**		
i.p. saline + water (control)	58.4/3.585	–
i.p. saline + KSMCT compared to control	59.6/2.577	–/0.188
i.p. 0.15 mg/kg DPCPX + KSMCT compared to control	66.8/6.304	–/1.260
i.p. 0.25 mg/kg DPCPX + KSMCT compared to control	61.5/5.565	–/0.470
i.p. 0.25 mg/kg DPCPX + water compared to control	53.3/7.440	–/0.771
**OPEN**		
i.p. saline + water (control)	95.8/2.932	–
i.p. saline + KSMCT compared to control	177.6/8.060	^****^/12.320
i.p. 0.15 mg/kg DPCPX + KSMCT compared to control	148.0/8.962	^****^/7.862
i.p. 0.25 mg/kg DPCPX + KSMCT compared to control	96.8/6.989	–/0.047
i.p. 0.25 mg/kg DPCPX + water compared to control	101.3/5.906	–/0.828
**CLOSED**		
i.p. saline + water (control)	145.9/3.482	–
i.p. saline + KSMCT compared to control	62.8/9.375	^****^/12.510
i.p. 0.15 mg/kg DPCPX + KSMCT compared to control	85.3/9.781	^****^/9.123
i.p. 0.25 mg/kg DPCPX + KSMCT compared to control	141.8/6.360	–/0.958
i.p. 0.25 mg/kg DPCPX + water compared to control	145.8/6.193	–/0.019
**Areas of EPM and different treatments (entries to the areas, number of entries, see Figure** **2B****)**	**Mean/**± **S.E.M**.	**Significance/*****q*****-value**
**CENTER**		
i.p. saline + water (control)	12.4/0.461	–
i.p. saline + KSMCT compared to control	12.6/0.324	–/0.573
i.p. 0.15 mg/kg DPCPX + KSMCT compared to control	12.9/0.479	–/1.147
i.p. 0.25 mg/kg DPCPX + KSMCT compared to control	12.6/0.596	–/0.573
i.p. 0.25 mg/kg DPCPX + water compared to control	13.3/0.590	–/2.006
**OPEN**		
i.p. saline + water (control)	7.0/0.327	–
i.p. saline + KSMCT compared to control	9.4/0.263	^**^/5.446
i.p. 0.15 mg/kg DPCPX + KSMCT compared to control	7.9/0.515	–/2.006
i.p. 0.25 mg/kg DPCPX + KSMCT compared to control	6.8/0.620	–/0.573
i.p. 0.25 mg/kg DPCPX + water compared to control	7.1/0.398	–/0.287
**CLOSED**		
i.p. saline + water (control)	6.4/0.324	–
i.p. saline + KSMCT compared to control	3.6/0.183	^***^/6.306
i.p. 0.15 mg/kg DPCPX + KSMCT compared to control	5.4/0.324	–/2.293
i.p. 0.25 mg/kg DPCPX + KSMCT compared to control	6.3/0.313	–/0.287
i.p. 0.25 mg/kg DPCPX + water compared to control	6.3/0.526	–/0.287

*Significance level was determined by Tukey's multiple comparisons test. DPCPX, 1,3-dipropyl-8-cyclopentylxanthine; EPM, elevated plus maze; i.p., intraperitoneally; KSMCT, ketone salt/KS + medium chain triglyceride/MCT (2.5 g/kg/day)*.

** *p < 0.01*,

*** p < 0.001, and

**** *p < 0.0001 level of significance*.

After KSMCT administration alone more entries to open and less entries to closed arms were observed (*p* < 0.01, *p* < 0.001, respectively; group 2) (Figure 2B), compared to control animals. Similarly to the effect on the time spent in the arms, i.p. injection of both doses of DPCPX (0.15 mg/kg and 0.25 mg/kg, group 3 and group 4, respectively) before the 7th administration of KSMCT decreased/abolished the effect of KSMCT on entries to arms (Figure 2B), while the higher dose of DPCPX (0.25 mg/kg, group 5) alone was ineffective in influencing the number of entries to arms (Figure 2B). None of the treatments changed the number of entries to the center (Figure 2B; Table 1).

### Effect of KSMCT and DPCPX on blood βHB and glucose levels, as well as on body weight

**Figure 3:**
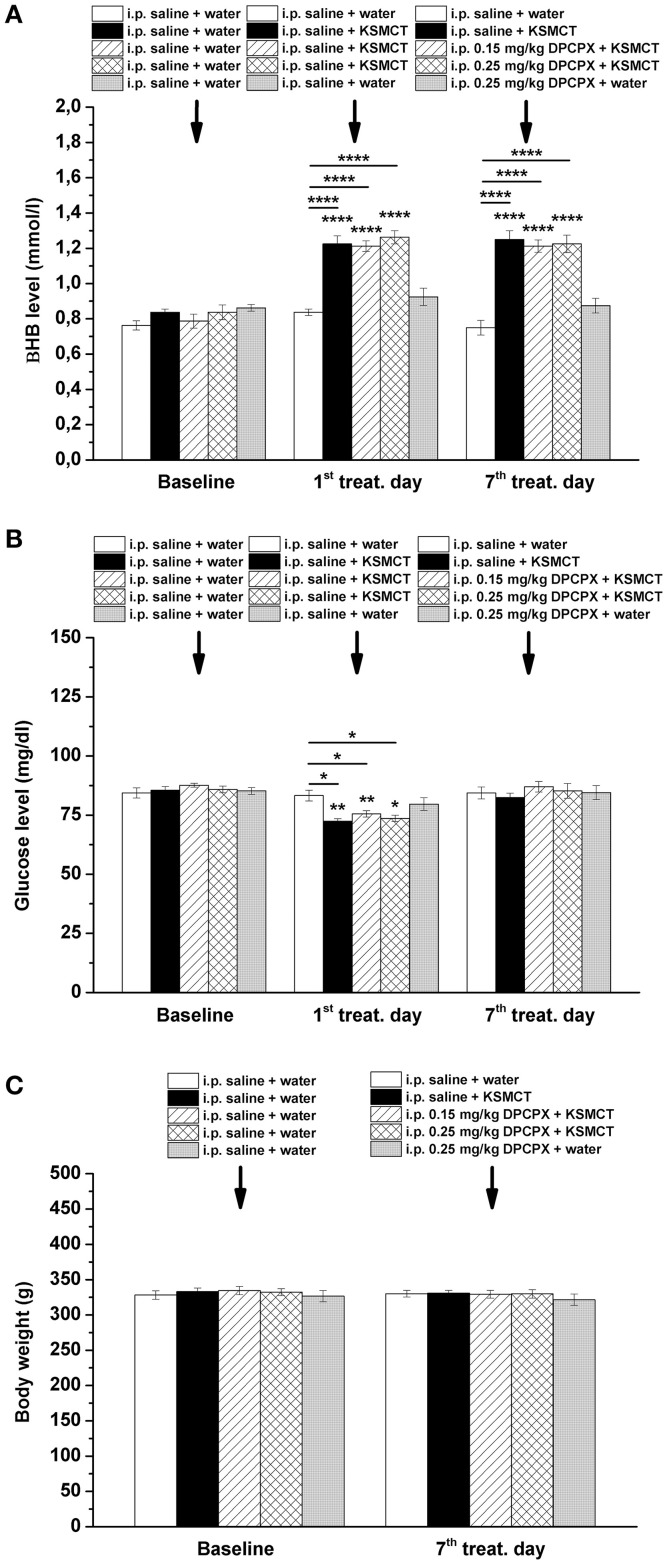
Effect of KSMCT (gavage, 2.5 g/kg/day, *n* = 8; i.p. saline + KSMCT) alone, KSMCT in combination with intraperitoneally (i.p.) injected two doses (0.15 mg/kg, *n* = 8; 0.25 mg/kg, *n* = 8) of DPCPX, as well as high dose of DPCPX (0.25 mg/kg, *n* = 8; DPCPX + water) on blood beta-hydroxybutyrate (βHB) **(A)** and, glucose **(B)** levels, as well as body weight **(C)** on the 1st and 7th day of gavage, compared to baseline and/or control (i.p. saline + water; *n* = 8) levels. All results are shown as means ± standard error of the mean (S.E.M.). βHB, blood beta-hydroxybutyrate; DPCPX, 1,3-Dipropyl-8-cyclopentylxanthine; i.p., intraperitoneally; KSMCT, ketone salt/KS + medium chain triglyceride/MCT; treat. Day, treatment day; ^*^p < 0.05; ^**^p < 0.01, and ^****^p < 0.0001 level of significance.

KSMCT gavage alone (group 2–4) significantly (*p* < 0.0001) increased the blood βHB levels after the 1st day and the 7th day of administration, compared to baseline and control levels (Figure 3A; Table 2). After combined application of KSMCT with lower (0.15 mg/kg) and higher (0.25 mg/kg) dose of DPCPX (group 3 and group 4), βHB levels were similar to concentrations observed after KSMCT alone (Figure 3A). Injection of high dose of DPCPX alone (group 5) did not have an effect on blood βHB levels (Figure 3A), compared to baseline and control levels (Table 2).

**Table 2:** Beta-hydroxybutyrate (βHB) levels after different treatments are presented (8 animals/group).

**Treatment days and different treatments (βHB level, mmol/l, see Figure 3A)**	**Mean/±S.E.M**.	**Significance/*q*-value**
**1st TREATMENT DAY**		
i.p. saline + water (control)	0.84/0.015	–
i.p. saline + KSMCT compared to control	1.23/0.045	^****^/10.260
i.p. saline + KSMCT compared to control	1.21/0.029	^****^/9.927
i.p. saline + KSMCT compared to control	1.26/0.038	^****^/11.250
i.p. saline + water compared to control	0.93/0.049	–/2.316
**7th TREATMENT DAY**		
i.p. saline + water (control)	0.75/0.042	–
i.p. saline + KSMCT compared to control	1.25/0.050	^****^/13.240
i.p. 0.15 mg/kg DPCPX + KSMCT compared to control	1.21/0.035	^****^/12.240
i.p. 0.25 mg/kg DPCPX + KSMCT compared to control	1.23/0.049	^****^/12.570
i.p. 0.25 mg/kg DPCPX + water compared to control	0.88/0.041	–/3.309
**BASELINE AND 1st TREATMENT DAY (1st tr. DAY)**		
i.p. saline + water (baseline)	0.76/0.026	–
i.p. saline + water (1st tr. day) compared to baseline	0.84/0.015	–/1.985
i.p. saline + water (baseline)	0.84/0.018	–
i.p. saline + KSMCT (1st tr. day) compared to baseline	1.23/0.045	^****^/12.240
i.p. saline + water (baseline)	0.79/0.039	–
i.p. saline + KSMCT (1st tr. day) compared to baseline	1.21/0.029	^****^/11.910
i.p. saline + water (baseline)	0.84/0.042	–
i.p. saline + KSMCT (1st tr. day) compared to baseline	1.26/0.038	^****^/13.240
i.p. saline + water (baseline)	0.86/0.018	–
i.p. saline + water (1st tr. day) compared to baseline	0.93/0.049	–/4.302
**BASELINE AND 7th TREATMENT DAY (7th tr. DAY)**		
i.p. saline + water (baseline)	0.76/0.026	–
i.p. saline + water (7th tr. day) compared to baseline	0.75/0.042	–/0.331
i.p. saline + water (baseline)	0.84/0.018	–
i.p. saline + KSMCT (7th tr. day) compared to baseline	1.25/0.050	^****^/12.900
i.p. saline + water (baseline)	0.79/0.039	–
i.p. 0.15 mg/kg DPCPX + KSMCT (7th tr. day) compared to baseline	1.21/0.035	^****^/11.910
i.p. saline + water (baseline)	0.84/0.042	–
i.p. 0.25 mg/kg DPCPX + KSMCT (7th tr. day) compared to baseline	1.23/0.049	^****^/12.240
i.p. saline + water (baseline)	0.86/0.018	–
i.p. 0.25 mg/kg DPCPX + water (7th tr. day) compared to baseline	0.88/0.041	–/2.978

*Significance level was determined by Tukey's multiple comparisons test. DPCPX: 1,3-dipropyl-8-cyclopentylxanthine; i.p.: intraperitoneally; KSMCT: ketone salt/KS + medium chain triglyceride/MCT (2.5 g/kg/day)*.

**** *p < 0.0001 level of significance*.

The blood glucose level significantly decreased on the 1st day of KSMCT gavage (group 2–4) (Figures 1, 3B), compared to baseline and control levels (Table 3). DPCPX alone (group 5) did not have an effect on blood glucose level. On the 7th day of KSMCT administration alone (group 2) and on the 7th day of KSMCT gavage, which were combined with i.p. injection of both doses of DPCPX (group 3 and group 4), as well as on the day of i.p. DPCPX alone (group 5), blood glucose levels did not change significantly (Figure 3B), compared to baseline and control levels (Table 3). Moreover, after administration of DPCPX in combination with KSMCT (group 3 and group 4) changes in glucose levels were similar to concentrations observed after KSMCT administration alone (group 2) (Figure 3B).

**Table 3:** Blood glucose levels after different treatments are presented.

**Treatment days and different treatments (glucose level, mg/dl, see Figure 3B)**	**Mean/±S.E.M**.	**Significance/*q*-value**
**1st TREATMENT DAY**		
i.p. saline + water (control)	83.25/2.242	–
i.p. saline + KSMCT compared to control	72.38/1.164	^*^/5.392
i.p. saline + KSMCT compared to control	75.50/1.349	^*^/5.144
i.p. saline + KSMCT compared to control	73.63/1.295	^*^/4.958
i.p. saline + water compared to control	79.63/2.659	–/1.797
**7th TREATMENT DAY**		
i.p. saline + water (control)	84.38/2.528	–
i.p. saline + KSMCT compared to control	82.38/1.851	–/0.992
i.p. 0.15 mg/kg DPCPX + KSMCT compared to control	87.00/2.204	–/1.302
i.p. 0.25 mg/kg DPCPX + KSMCT compared to control	85.25/3.109	–/0.434
i.p. 0.25 mg/kg DPCPX + water compared to control	84.50/2.988	–/0.062
**BASELINE AND 1st TREATMENT DAY (1st tr. DAY)**		
i.p. saline + water (baseline)	84.35/2.179	–
i.p. saline + water (1st tr. day) compared to baseline	83.25/2.242	–/0.558
i.p. saline + water (baseline)	85.50/1.592	–
i.p. saline + KSMCT (1st tr. day) compared to baseline	72.38/1.164	^**^/5.950
i.p. saline + water (baseline)	87.63/0.800	–
i.p. saline + KSMCT (1st tr. day) compared to baseline	75.50/1.349	^**^/5.702
i.p. saline + water (baseline)	85.88/1.419	–
i.p. saline + KSMCT (1st tr. day) compared to baseline	73.63/1.295	^*^/5.516
i.p. saline + water (baseline)	85.21/1.398	–
i.p. saline + water (1st tr. day) compared to baseline	79.63/2.659	–/2.355
**BASELINE AND 7th TREATMENT DAY (7th tr. DAY)**		
i.p. saline + water (baseline)	84.35/2.179	–
i.p. saline + water (7th tr. day) compared to baseline	84.38/2.528	–/0.310
i.p. saline + water (baseline)	85.50/1.592	–
i.p. saline + KSMCT (7th tr. day) compared to baseline	82.38/1.851	–/0.992
i.p. saline + water (baseline)	87.63/0.800	–
i.p. 0.15 mg/kg DPCPX + KSMCT (7th tr. day) compared to baseline	87.00/2.204	–/1.302
i.p. saline + water (baseline)	85.88/1.419	–
i.p. 0.25 mg/kg DPCPX + KSMCT (7th tr. day) compared to baseline	85.25/3.109	–/0.434
i.p. saline + water (baseline)	85.21/1.398	–
i.p. 0.25 mg/kg DPCPX + water (7th tr. day) compared to baseline	84.50/2.988	–/0.062

*Significance level was determined by Tukey's multiple comparisons test (8 animals/groups). DPCPX, 1,3-dipropyl-8-cyclopentylxanthine; i.p., intraperitoneally; KSMCT, ketone salt/KS + medium chain triglyceride/MCT (2.5 g/kg/day)*.

* p < 0.05 and

** *p < 0.01 level of significance*.

The body weight did not change significantly in either group (group 1–5), compared to baseline and control levels (Figure 3C).

## Discussion

In this study we demonstrated that inhibition of A_1_Rs by i.p. DPCPX dose-dependently decreased/abolished the anxiolytic effect of exogenous ketone supplement KSMCT, in WAG/Rij rats. In addition, we confirmed our previous results showing that sub-chronic administration of KSMCT alone administered by intragastric gavage has anxiolytic effect in WAG/Rij rats (Ari et al., 2016).

Anxiety disorders (e.g., phobias and panic disorder) are the most prevalent mental disorders, which disorders can be associated with disability, psychiatric morbidity, as well as mortality and can be characterized by excessive fear, avoidance behaviors, and anxiety (Stahl, 2003; Li, 2012; Mula, 2013). The exact cause and pathomechanism(s) of anxiety disorders is poorly understood. However, it is widely accepted that, among others, the amygdala, the hippocampus and the ventromedial prefrontal cortex are involved in the neurobiology of anxiety disorders. It has also been demonstrated that deficits in the extinction of conditioned fear and overgeneralization of conditioned fear have a role in the appearance of anxiety disorders (Milad et al., 2014; Dunsmoor and Paz, 2015; Rigoli et al., 2016). In addition, it was also suggested that overactivity in limbic regions (e.g., insula and amygdala) and aberrant functional connectivity of brain regions implicated in anxiety disorders may contribute to the development of anxiety disorders (Stahl, 2003; Craske and Stein, 2016).

Although very little is known about the link between ketone-induced changes in anxiety disorders, it has been suggested previously that ketone supplementation may be a potential therapeutic intervention in treatment of anxiety disorders (Masino et al., 2012; Ari et al., 2016). It has been demonstrated that ketogenic diet and ketone bodies may enhance the GABAergic effects by GABA_A_ receptors, attenuate extracellular glutamate release and increase adenosine level (Grillon et al., 2006; Norberg et al., 2008; Masino et al., 2012; McNally and Hartman, 2012), which processes may modulate anxiety level. Indeed, it has also been suggested that not only serotoninergic, glutamatergic, and GABAergic, but also the adenosinergic system of different brain areas are implicated in mechanisms of anxiety (e.g., hippocampus and amygdala). Therefore, serotonin transporters/receptors, N-methyl-D-aspartate receptors, GABA receptors and adenosine receptors are all considered potential targets in the treatment of anxiety disorders (Florio et al., 1998; Kakui et al., 2009; Li, 2012; Sankar, 2012).

Exogenous ketone supplement-induced elevation in βHB and AcAc concentration may enhance brain energy metabolism and neurotransmitter balance while preserving physiological and neurometabolic processes under conditions of extreme oxidative stress (Yudkoff et al., 2007; D'Agostino et al., 2013; Poff et al., 2014, 2015; Ari et al., 2016) or persistent molecular pathology (Ciarlone et al., 2016). Indeed, it has been demonstrated that exogenous ketone supplements (independent of diet), such as KE, KS and KSMCT, may be a promising alternative method (as a metabolic-based therapy) for different disorders, such as Alzheimer's disease, anxiety, epilepsy and cancer (D'Agostino et al., 2013; Newport et al., 2015; Poff et al., 2015; Ari et al., 2016). Recent studies indicate that anti-anxiety and anti-epileptic effects of exogenous ketone supplements, such as KSMCT, may be in relation to their ability to increase the level of blood βHB, which supports the applicability of nutritional ketosis as a therapeutic tool to suppress both anxiety and absence epileptic seizures (Groomes et al., 2011; Azzam and Azar, 2013; Ari et al., 2016; Kovács et al., 2017). Increased level of ketone bodies may change the activity of different neurotransmitter systems, such as GABAergic and adenosinergic system: high level of ketosis/βHB may evoke changes in metabolic pathways, which lead to increased levels of both gamma-aminobutyric acid (GABA) and adenosine (Sharma et al., 2015; Ciarlone et al., 2016). Our present study demonstrates that KSMCT increased the βHB level in parallel with a decrease in anxiety level, whereas DPCPX in combination with KSMCT decreased/abolished the effect of KSMCT administration on anxiety level, but did not change the KSMCT administration-induced elevation in blood βHB level. In addition, DPCPX alone did not change blood βHB level. These results suggest that elevated level of βHB may have a role in the KSMCT administration induced anti-anxiety effect, potentially, *via* βHB-generated increase in adenosine concentration and through A_1_Rs.

It has been demonstrated that not only A_1_R-knockout mice, but also adenosine A_2A_ receptor/A_2A_R-knockout mice showed enhanced level of anxiety, suggesting that both adenosine receptor types influence anxiety behavior (Ledent et al., 1997; Johansson et al., 2001; Giménez-Llort et al., 2002; Cunha et al., 2008). Indeed, our results support the observations demonstrating the alleviating effect of adenosine on anxiety by means of A_1_Rs. Interestingly, the inhibition of adenosine receptors by caffeine promotes anxious behavior, which influence was not shared by selective A_2A_R antagonists (Klein et al., 1991; El Yacoubi et al., 2000). Selective activation (e.g., by an A_1_R agonist *N*^6^-cyclopentyladenosine/CPA) and inhibition (e.g., by an A_1_R antagonist 8-cyclopentyltheophylline/CPT) of centrally located A_1_Rs evoked anxiolytic and anxiogenic effects, respectively, on EPM (Jain et al., 1995; Florio et al., 1998), while A_2A_R receptor agonists and antagonists had no effect on anxiety behavior (Jain et al., 1995; El Yacoubi et al., 2000). In previous studies the administration of DPCPX (0.05 and 0.5 mg/kg) alone did not change the level of anxiety (Jain et al., 1995), whereas DPCPX prevented the anxiolytic effect of CPA (Jain et al., 1995). Similarly to this previous study, we also demonstrated that DPCPX alone was not able to influence anxiety level, but abolished the KSMCT-evoked anxiolytic effect on EPM.

Expression of adenosine receptors in the brain areas implicated in anxiety disorders (e.g., amygdala and hippocampus) and the uneven distribution of these receptors and adenosine levels were demonstrated in the CNS (Kovács et al., 2011b). This observation implies region-dependent roles of adenosine in the brain, such as the modulation of pathophysiology of anxiety disorders. In addition, KSMCT administration decreased not only the anxiety level (Ari et al., 2016), but also the absence epileptic activity in WAG/Rij rats (Kovács et al., 2017). Interestingly, influence of KSMCT on both anxiety level and absence epileptic activity were abolished by DPCPX (Kovács et al., 2017), suggesting not only the neuromodulatory role of A_1_Rs in the anxiolytic and anti-epileptic effect of KSMCT, but also the applicability of drugs effective on A_1_Rs in the treatment of absence epilepsy and anxiety (Brandt and Mula, 2016). As to the mechanism of action, it is widely accepted that endogenous adenosine mediates physiological and pathophysiological effects through A_1_Rs, by which adenosine depresses synaptic transmission and neuronal activity (e.g., by activation of both G protein-gated inwardly rectifying potassium channels and ATP-sensitive potassium/K_ATP_ channels, Dunwiddie and Masino, 2001; Kovács et al., 2011b, 2015). It has been demonstrated that ketone bodies increase the intracellular level of ATP (Achanta and Rae, 2017), enhance the ATP efflux through pannexin channels and increase the extracellular adenosine level through metabolism of ATP by ectonucleotidases. Adenosine, by activation of A_1_Rs, opens the K_ATP_ channels and evokes neuronal hyperpolarization and a decrease in neuronal firing of central neurons (Ma et al., 2007; Kawamura et al., 2010; Achanta and Rae, 2017; Simeone et al., 2017). Therefore, A_1_Rs may inhibit the release of excitatory neurotransmitters in the CNS (Ribeiro, 1995), which neurotransmitters (e.g., glutamate) are implicated in the pathophysiology of both anxiety and absence epilepsy (Snead, 1995; Coenen and Van Luijtelaar, 2003; Li, 2012). Indeed, βHB inhibits presynaptic release of glutamate and, as a consequence, attenuates excess neuronal excitability (Juge et al., 2010). Therefore, it is possible that activation of A_1_Rs may decrease the glutamate-induced hyperexcitability of brain structures (Masino and Geiger, 2008) implicated in anxiety disorders (such as orbitofrontal/prefrontal cortex, thalamus and striatum, Stahl, 2003) and absence epilepsy (e.g., somatosensory cortex/cortical focus of absence epilepsy genesis, Coenen and Van Luijtelaar, 2003), at least in WAG/Rij rats. Therefore, the KSMCT-evoked increase in ketone body level (directly and/or by adenosine *via* its receptors), may modulate other neurotransmitter systems implicated in anxiety. Thus, it is possible that KSMCT exerts its effect on anxiety through not only adenosinergic and glutamatergic but also serotoninergic and GABAergic systems (Grillon et al., 2006; Norberg et al., 2008; Li, 2012; Ciarlone et al., 2016). However, further studies are needed to determine the exact role of different neurotransmitter systems in KSMCT induced anxiolytic effect.

Based on the present results, putative effect of KSMCT-induced changes in glucose concentration on anxiety levels can be excluded, because significant effects on glucose levels was only demonstrated after the first application of KSMCT. Similarly, the body weight decrease-evoked effects by enhanced metabolism of fat tissue/free fatty acids and, as a consequence, increased βHB level did not have a role in the KSMCT-generated effects, because body weight did not change significantly after KSMCT administration (Cahill, 2006). However, the role of GABAergic and other transmitter systems, such as serotoninergic and glutamatergic, or other factors, such as changes in blood glucose levels cannot be excluded entirely and requires further investigation.

## Conclusion

Based on these results we can conclude that administration of the exogenous ketone supplement, KSMCT, may be a potential therapeutic approach in the treatment of therapy-resistant types of anxiety disorders by working, at least partially through the adenosinergic system. Inducing nutritional ketosis by exogenous ketogenic supplements allows for a rapid and sustained dietary ketosis independent of dietary restriction. In addition, our present study provided us an opportunity to investigate new aspects of the pathophysiology of anxiety, which remains poorly understood. However, further efforts are needed to elucidate all contributing neurometabolic, adenosinergic- and other neurotransmitter system-induced signaling mechanisms of exogenous ketone supplements on anxiety behavior.

## Author contributions

ZK: conception and design of experiments, data collection, interpretation of data, and writing manuscript; DD: interpretation of data, writing manuscript; CA: conception of experiments, data analysis, writing manuscript.

### Conflict of interest statement

International Patent # PCT/US2014/031237, University of South Florida, D. P. D'Agostino, S. Kesl, P. Arnold, “Compositions and Methods for Producing Elevated and Sustained Ketosis.” Non-provisional patent #62289749, University of South Florida, C. Ari, D. P. D'Agostino, “Exogenous ketone supplements for reducing anxiety-related behavior.” DD and CA are co-owners of the company Ketone Technologies LLC. These interests have been reviewed and managed by the University in accordance with its Institutional and Individual Conflict of Interest policies. The other author declares that the research was conducted in the absence of any commercial or financial relationships that could be construed as a potential conflict of interest.

## Abbreviations

A_1_R: adenosine A_1_ receptor
A_2A_R: adenosine A_2A_ receptor
AcAc: acetoacetate
ATP: adenosine triphosphate
βHB: beta-hydroxybutyrate
CPA: *N*^6^-cyclopentyladenosine
DMSO: dimethyl sulfoxide
DPCPX: 1,3-dipropyl-8-cyclopentylxanthine
EPM: elevated plus maze
GABA: gamma-aminobutyric acid
i.p.: intraperitoneal
KE: ketone ester
KS: ketone salt
KSMCT: ketone salt/KS + medium chain triglyceride/MCT
MCT: medium chain triglyceride
WAG/Rij: Wistar Albino Glaxo/Rijswijk.
